# Different Cre systems induce differential microRNA landscapes and abnormalities in the female reproductive tracts of Dgcr8 conditional knockout mice

**DOI:** 10.1111/cpr.12996

**Published:** 2021-01-26

**Authors:** Yeon Sun Kim, Seung Chel Yang, Mira Park, Youngsok Choi, Francesco J. DeMayo, John P. Lydon, Hye‐Ryun Kim, Hyunjung Jade Lim, Haengseok Song

**Affiliations:** ^1^ Department of Biomedical Science CHA University Seongnam Korea; ^2^ Department of Stem Cell and Regenerative Biotechnology Konkuk University Seoul Korea; ^3^ Department of Reproductive and Developmental Biology Laboratory National Institute of Environmental Health Sciences Research Triangle Park NC USA; ^4^ Department of Molecular and Cellular Biology and Center for Reproductive Medicine Baylor College of Medicine Houston TX USA; ^5^ Department of Veterinary Medicine, School of Veterinary Medicine Konkuk University Seoul Korea; ^6^ Present address: Division of reproductive sciences Department of Pediatrics Cincinnati Children’s Hospital OH USA

**Keywords:** *Dgcr8*, *Dgcr8* conditional knockout mice, female reproductive tract, MicroRNAs, multiple Cre systems, Phenotypes

## Abstract

**Objectives:**

The female reproductive tract comprises several different cell types. Using three representative Cre systems, we comparatively analysed the phenotypes of *Dgcr8* conditional knockout (cKO) mice to understand the function of *Dgcr8*, involved in canonical microRNA biogenesis, in the female reproductive tract.

**Materials and Methods:**

*Dgcr8* ^f/f^ mice were crossed with *Ltf* ^icre/+^, *Amhr2* ^cre/+^ or *PR* ^cre/+^ mice to produce mice deficient in *Dgcr8* in epithelial (*Dgcr8* ^ed/ed^), mesenchymal (*Dgcr8* ^md/md^) and all the compartments (*Dgcr8* ^td/td^) in the female reproductive tract. Reproductive phenotypes were evaluated in *Dgcr8* cKO mice. Uteri and/or oviducts were used for small RNA‐seq, mRNA‐seq, real‐time RT‐PCR, and/or morphologic and histological analyses.

**Result:**

*Dgcr8* ^ed/ed^ mice did not exhibit any distinct defects, whereas *Dgcr8* ^md/md^ mice showed sub‐fertility and oviductal smooth muscle deformities. *Dgcr8* ^td/td^ mice were infertile due to anovulation and acute inflammation in the female reproductive tract and suffered from an atrophic uterus with myometrial defects. The microRNAs and mRNAs related to immune modulation and/or smooth muscle growth were systemically altered in the *Dgcr8* ^td/td^ uterus. Expression profiles of dysregulated microRNAs and mRNAs in the *Dgcr8* ^td/td^ uterus were different from those in other genotypes in a Cre‐dependent manner.

**Conclusions:**

*Dgcr8* deficiency with different Cre systems induces overlapping but distinct phenotypes as well as the profiles of microRNAs and their target mRNAs in the female reproductive tract, suggesting the importance of selecting the appropriate Cre driver to investigate the genes of interest.

## Graphical abstract

We comparatively analysed the phenotypes of *Dgcr8* conditional knockout (cKO) mice to understand the function of *Dgcr8*, involved in canonical microRNA biogenesis, in the female reproductive tract. Because uterine is composed of various cell types, we generated various *Dgcr8* cKO mice using representative Cre mice (*Ltf^icre/+^*, *Amhr2^cre/+^*, and *PR^cre/+^* mice) to dissect functions of *Dgcr8* depending on uterine cell type‐specific. Each *Dgcr8* cKO mice shows distinct and overlapping phenotypes as well as microRNAs and their target mRNA landscapes. These results suggest the importance of selecting the appropriate Cre driver to investigate genes of interest

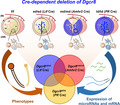

## 1 INTRODUCTION

MicroRNAs (miRNAs) are evolutionarily conserved small non‐coding RNAs that function in RNA silencing and post‐transcriptional regulation of gene expression. miRNAs also regulate various cellular pathways necessary for the development and proper functions of organs, such as the oviduct and uterus, in the female reproductive tract.^1^, ^2^ DGCR8 is an RNA‐binding protein that works with DROSHA to produce precursor miRNA in the nucleus, while DICER generates mature miRNAs and endogenous small interfering RNAs in the cytoplasm.^3^ To study the function of miRNAs, especially canonical miRNAs in the female reproductive tract, we generated *Dgcr8* ^f/f^; *progesterone receptor* (*PR*)*^cre/+^* (*Dgcr8* ^td/td^) mice and reported that canonical miRNAs are essential for uterine morphogenesis and physiology, including natural immune modulation.^4^ *PR* ^cre/+^ (*PR*‐Cre) mice have been mostly used to study uterine biology during pregnancy and various diseases. However, *PR*‐Cre inactivates genes not only in the female reproductive tract but also in other progesterone‐responsive organs, including the ovary, pituitary gland and mammary gland.^5^ In the uterus, PR is spatiotemporally expressed in all the major uterine compartments: myometrium, stroma, and epithelium. Furthermore, PR is also expressed in immune cells, such as natural killer (NK) cells,^6^ macrophages,^7^ dendritic cells^8^ and T cells,^9^ suggesting that *PR*‐Cre may affect various immune cells as well as all the major uterine cells in a spatiotemporal manner.

In addition to *PR*‐Cre, other Cre mice with unique purposes are currently available for conditionally inactivating gene(s) of interest in the female reproductive tract, especially in the uterus. *Anti‐mullerian hormone receptor type 2* (*Amhr2*)‐Cre mice are mainly used to target genes in stromal and myometrial compartments of the uterus and oviduct as well as of the ovary.^10^ Temporally, Cre action starts from midgestational embryo development (embryonic day 12.5) under the control of the *Amhr2* promoter in *Amhr2*‐Cre mice. Recently, other Cre mice, such as *lactoferrin* (*Ltf*)‐iCre, s*mall proline‐rich protein 2f* (*Sprr2f*)‐Cre, and *Wnt family member 7a* (*Wnt7a*)‐Cre, were generated to target genes in the epithelial compartment. The spatiotemporal actions of each Cre on the uterine epithelium are unique. Although *Wnt7a*‐Cre is expressed throughout the epithelium of the prenatal Müllerian tract,^11^ *Ltf* and *Sprr2f* are not expressed in the immature mouse uterus, but robustly expressed in the uterine epithelium of adult mice.^12^, ^13^ *Ltf* and *Sprr2f*, well‐known oestrogen‐responsive genes, are expressed not only in the uterine but also in the oviductal epithelium after puberty.^13^ Collectively, the spatiotemporal modes of each Cre system may provide diverse reproductive phenotypes. Thus, insights into the mode(s) of actions of multiple Cre systems are required to precisely delineate the functions of genes of interest in a cell type‐specific manner in the female reproductive tract.

## 2 MATERIALS AND METHODS

### 2.1 Animals and genotyping of *Dgcr8* cKO mice

All mice used in this study were maintained in accordance with the policies of the Institutional Animal Care and Use Committee (IACUC170174) of CHA University. *Dgcr8* ^f/f^ mice were initially generated and provided by Dr Elaine Fuchs.^14^ *PR‐*Cre, *Amhr2*‐Cre and *Ltf*‐iCre mice were generously provided by Dr Francesco DeMayo,^5^ Dr Richard Behringer^10^ and Dr Sudhansu K. Dey,^12^ respectively. Genotyping PCR was performed using specific primers (Table S1) and genomic DNA extracts from mouse tail biopsies.

### 2.2 Fertility analysis and preimplantation embryo culture

*Dgcr8* ^td/td^ mice have an anestrous cycle; therefore, to induce ovulation, *Dgcr8* ^td/td^ mice were administered intraperitoneal (IP) injections of 5 IU PMSG (Sigma‐Aldrich, St. Louis, MO, USA) for 48 hours, followed by IP injections of 5 IU hCG (Sigma‐Aldrich). *Dgcr8* ^td/td^ mice were then bred with wild‐type fertile males*,* and pregnancy was evaluated by the presence of a vaginal plug the next morning. The other *Dgcr8* cKO mice were naturally mated. The 2‐cell embryos and/or fragmented oocytes were flushed from the oviducts on day 2 of pregnancy (Day 2).

### 2.3 RNA extraction, reverse transcription‐PCR (RT‐PCR) and real‐time RT‐PCR

Total RNA was extracted from the mouse uterus using TRIzol Reagent (Invitrogen Life Technologies, San Diego, CA, USA) according to the manufacturer's protocols. First‐strand cDNA was synthesized from 1 μg of total RNA using M‐MLV reverse transcriptase (Promega, Madison, WI, USA) and RNasin Ribonuclease Inhibitor (Promega). For quantification of expression levels, real‐time RT‐PCR was performed using iQ™ SYBR Green Supermix (Bio‐Rad, Hercules, CA, USA) on a BIO‐RAD iCycler as previously described.^4^ The synthesized cDNA was utilized for PCR and real‐time RT‐PCR with specific primers (Table S1).

### 2.4 Tissue collection and histological analysis

Female reproductive organs were dissected and fixed in 4% paraformaldehyde (PFA) for histology or snap‐frozen for RNA and/or protein preparation. Tissues were embedded in paraplast (Leica Biosystems, St. Louis LLC, Diemen, the Netherlands). Sections were cut at 5 μm and stained with haematoxylin and eosin (H&E) (Sigma‐Aldrich), and observed by light microscopy.

### 2.5 Immunofluorescence

Sections were subjected to antigen retrieval in 10 mM sodium citrate buffer (pH 6.0) for 20 min. Non‐specific staining was blocked using protein block serum (Dako, Carpinteria, CA, USA). Sections were then incubated with primary smooth muscle actin (α‐SMA) (Abcam, Cambridge, UK, 1:100) and acetylated tubulin (Sigma‐Aldrich, 1:200), E‐cadherin (Cell Signaling, Danvers, MA, USA, 1:200), Desmin (Santa Cruz, Dallas, TX, USA, 1:200) and CD45 (Novus, Centennial, CO, USA, 1:200) antibody. In addition, sections were stained with TO‐PRO‐3‐iodide (Invitrogen) or DAPI (Thermo, Waltham, MA, USA).

### 2.6 Library preparation for small RNA sequencing (RNA‐seq) and miRNA expression analysis

For control and test RNAs, the construction of the library was performed using the NEBNext Multiplex Small RNA Library Prep kit (New England BioLabs, Inc, USA) according to the manufacturer's instructions. Briefly, for library construction, total RNA from each sample was used 1 μg to ligate the adapters, and then, cDNA was synthesized using reverse transcriptase with adaptor‐specific primers. To quantify the miRNA expression levels, total RNA (1 μg) was converted to cDNA, and real‐time RT‐PCR (50 ng of cDNA) was performed following the protocol from the HB miR Multi Assay Kit (Heim Biotek, Gyeonggi‐do, Korea).

### 2.7 mRNA‐Seq and data analyses

Uteri were dissected and snap‐frozen. Quant‐3’ mRNA‐seq was initially performed using total uterine RNA pooled by genotype (n = 2‐3 pools per genotype; 3 mice per pool; Ebiogen, Seoul, Korea). High‐throughput sequencing was performed as single‐end 75 sequencing using NextSeq 500 (Illumina, Inc, USA). Gene classification was based on searches performed using GSEA software (Gene Set Enrichment Analysis)^15^ and QuickGO (https://www.ebi.ac.uk/QuickGO/), and the target genes of miRNAs were identified using miRmap (miRmap score ≥ 90; https://mirmap.ezlab.org/).

### 2.8 Cell culture, transfection and luciferase assay

293T cells were grown in high glucose DMEM supplemented with 10% FBS, penicillin at 37°C, and 5% CO_2_. 293T cells were transiently transfected using Lipofectamine 3000 (Invitrogen Life Technologies). Transfection of each 3’ UTR into a pmirGLO basic luciferase reporter vector (Promega; 300 ng) and each mimic miRNA (Bioneer, Daejeon, Korea; 25 pg) was performed in a 24‐well plate. Luciferase assay was performed at 48 hours post‐transfection. Renilla luciferase and firefly luciferase activities were analysed using the Dual‐Luciferase® Reporter Assay System (Promega) following the manufacturer's instructions. The firefly luciferase activities were normalized using Renilla luciferase activity.

### 2.9 Statistical analysis

All values represent the mean ± standard deviation. Statistical analyses were performed using the unpaired Student's *t* tests and *P* < 0.05 was considered statistically significant for more than 3 groups.

## 3 RESULTS

### 3.1 Multiple Cre systems effectively delete *Dgcr8* in the female reproductive tract

**FIGURE 1:**
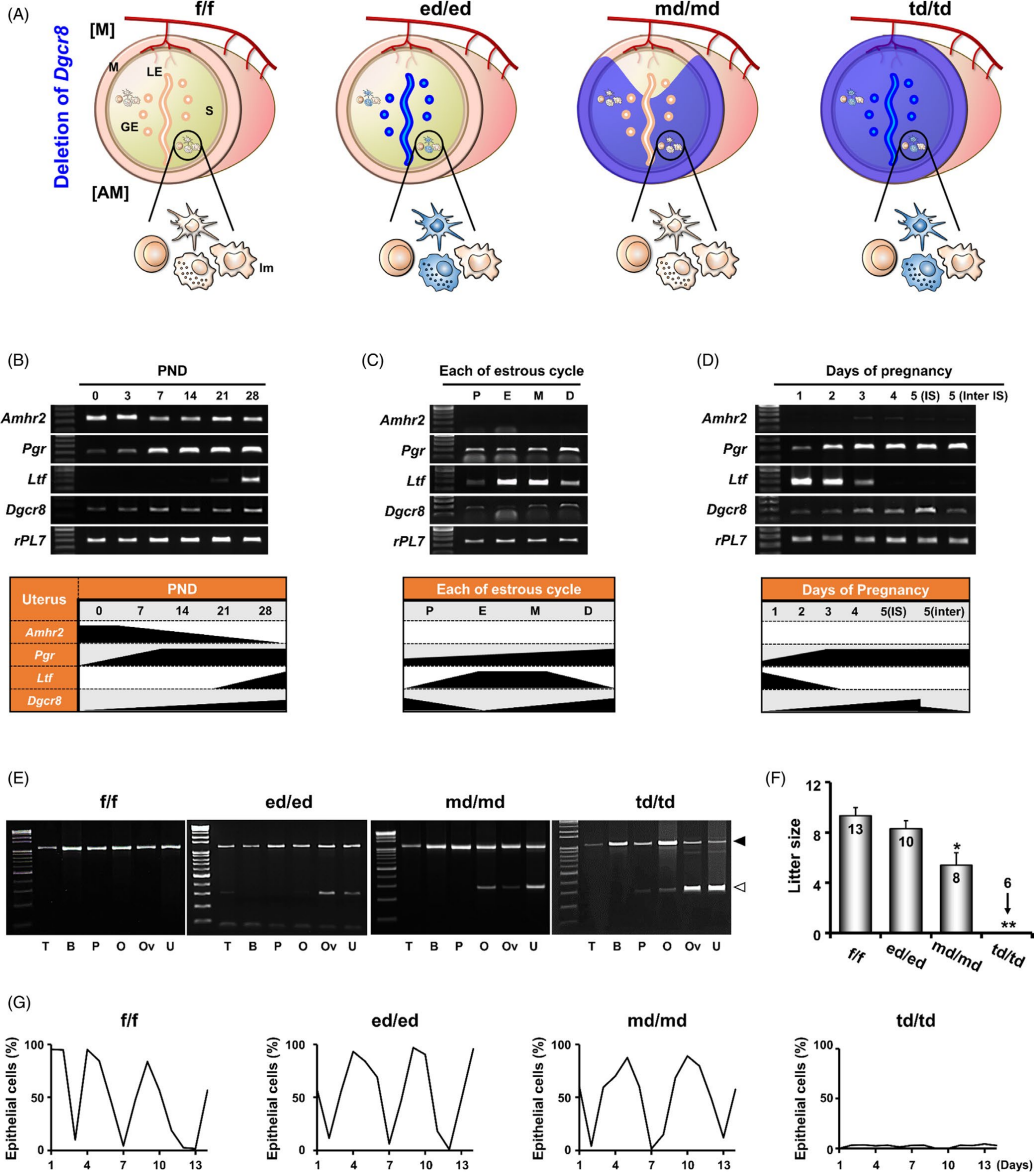
Conditional deletion of *Dgcr8* in female reproductive tracts by various uterine Cre systems and fertility tests in *Dgrc8* cKO mice. A, A schematic diagram summarizing the expression of various Cre systems. GE, Glandular epithelium; LE, Luminal epithelium; S, Stroma; M, Myometrium; [M], Mesometrium; [AM], Anti‐mesometrium; lm, Immune cell. (B‐D) RT‐PCR analyses of *Amhr2*, *Pgr, Ltf* and *Dgcr8* were performed using total RNA extracted from the mouse uterus at various postnatal days (PND), during the oestrous cycle, and early pregnancy (n = 5 per group). P, Proestrus; E, Oestrus; M, Metestrus; D, Dioestrus; IS, Implantation site; Inter IS, Inter‐implantation site. E, Representative images of PCR results with the genomic DNA of various tissues. Black and white arrowheads indicate PCR products for inclusion (1085 bp) and deletion (262 bp) of *Dgcr8* exon 3, respectively. T, Tail; B, Brain; P, Pituitary gland; O, Ovary; Ov, Oviduct; U, Uterus. F, Litter size in *Dgcr8* ^f/f^ and *Dgcr8* cKO mice. Numbers in bars indicate the number of mice examined in each group. Unpaired Student's *t* test, *P < 0.05, **P < 0.01. G, Representative graphs to demonstrate changes in epithelial cells/total cells (%) obtained by a vaginal smear method for two weeks (n = 4 per genotype)

The uterine cell type‐specific actions of the three representative Cre systems used in this study were summarized based on previous reports (Figure 1A). To understand the temporal activity of Cre drivers in the female reproductive tract, the expression profiles of *Amhr2*, *Pgr and Ltf* were first examined during postnatal days (PND), oestrous cycle and early pregnancy (Figure 1B‐D and Figure S1). RT‐PCR results showed that *Amhr2* is highly expressed in the developing uterus at PND 0, but maintained at undetectable levels during the oestrous cycle and early pregnancy. *Pgr* expression was very low at PND 0 and 3 but increased after PND 7 in the uterus. *Ltf* mRNAs were detected in the mouse uterus at PND 28 (4 weeks of age). During the oestrous cycle*, Pgr* and *Ltf* showed stage‐specific expression patterns. During early pregnancy, *Ltf* expression with a peak level on Day 1 gradually decreased onward, whereas *Pgr* expression was low on Days 1 and 2, followed by substantial increases on Days 3‐5.

To validate the actions of each Cre system in the female reproductive tract of *Dgcr8* cKO mice, *Dgcr8* ^f/f^ mice were crossed with *Ltf*‐iCre, *Amhr2*‐Cre and *PR*‐Cre mice to produce *Dgcr8* ^ed/ed^ (epithelium‐specific), *Dgrc8* ^md/md^ (mesenchyme‐specific) and *Dgrc8* ^td/td^ (all the major uterine cell types) mice, respectively. Cre‐mediated deletion of exon 3 of the *Dgcr8* allele produced a 262 bp PCR product (white arrowhead), whereas a floxed allele resulted in a 1085 bp product (black arrowhead) (Figure 1E). Consistent with a previous report^12^, ^16^ that *Ltf* is expressed in the epithelium of the oviduct and uterus and in some immune cells, PCR results using genomic DNA of tissues from *Dgcr8* ^ed/ed^ mice showed a deletion of exon 3 in the oviduct and uterus, but not in other tissues. *Dgcr8* ^md/md^ mice showed deletion of *Dgcr8* only in the ovary, oviduct and uterus, among all the tested tissues. *PR*‐Cre activity was detected not only in the female reproductive tract but also in the pituitary of *Dgcr8* ^td/td^ mice.

### 3.2 *Dgcr8* cKO mice crossed with different Cre systems show a distinct spectrum of fertility

To compare the fertility of *Dgcr8* cKO female mice with different Cre systems, they were mated with mature fertile males for 8‐10 weeks (Figure 1F). *Dgcr8* ^td/td^ female mice never produced any litter, as we previously reported.^4^ However, *Dgcr8* ^md/md^ mice were sub‐fertile and *Dgcr8* ^ed/ed^ mice were normal with respect to the number of pups produced. By monitoring oestrous cycles with daily vaginal smears over a 2‐week period, we observed that *Dgcr8* ^td/td^ female mice were anestrus*,* whereas *Dgcr*8^md/md^ and *Dgcr8* ^ed/ed^ mice exhibited regular 4‐5 day oestrous cyclicity similar to that of *Dgcr8* ^f/f^ control mice (Figure 1G).

### 3.3 *Dgcr8* deficiency in the oviduct affects quality of ovulated oocytes followed by fertilization in *Dgcr8* ^md/md^ mice

**FIGURE 2:**
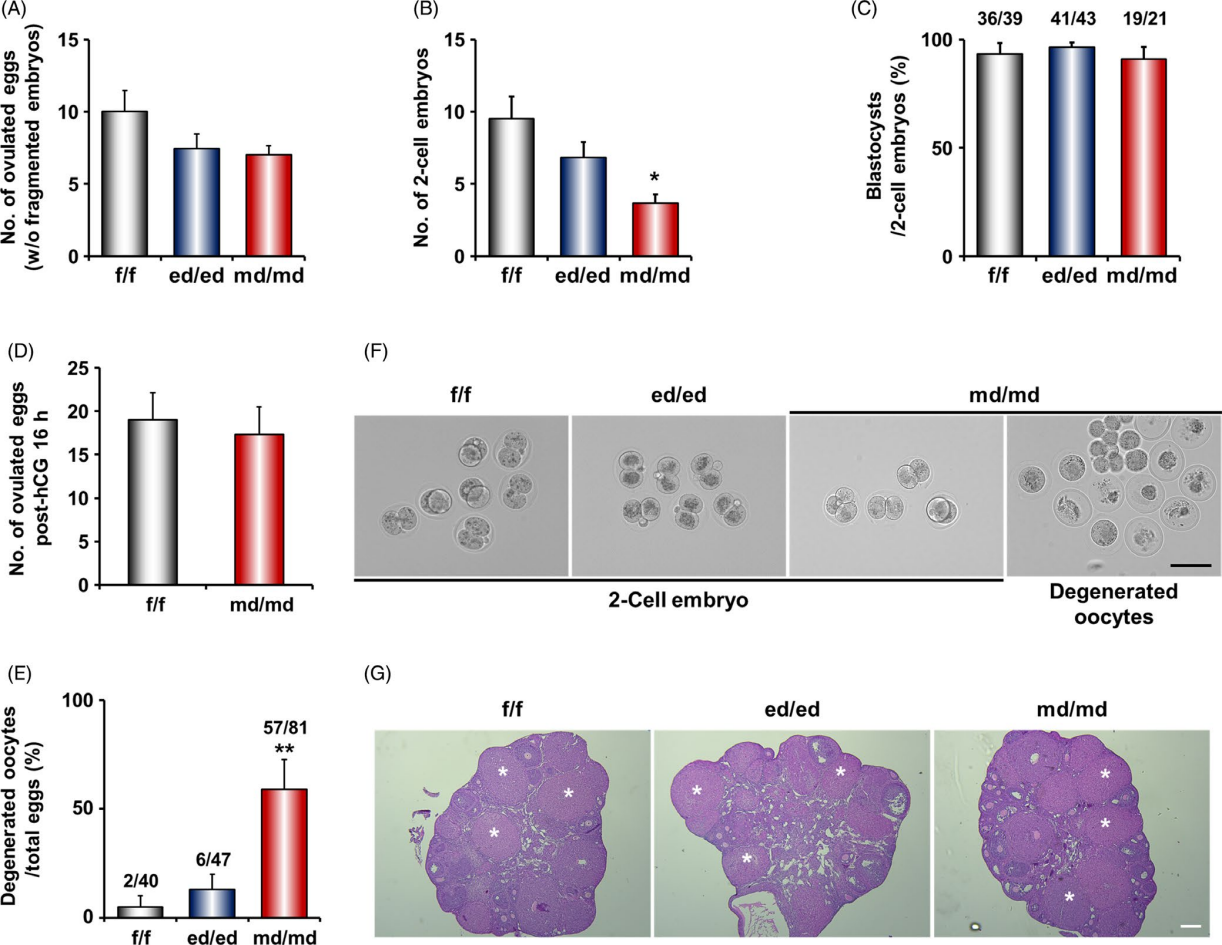
The comparison of ovulation and embryo development rate in *Dgcr8* cKO mice on Day 2. (A‐C) 8‐week‐old *Dgcr8* ^f/f^ and *Dgcr8* cKO mice were mated with wild‐type fertile males. Ovulation and embryo development are comparable between *Dgcr8* ^f/f^ and cell type‐specific *Dgcr8* cKO mice (n = 4‐6 per genotype). D, The number of ovulated eggs via superovulation in *Dgcr8* ^f/f^ and *Dgcr8* ^md/md^ mice (n = 12‐15 per genotype). E, The percentage of degenerated oocytes in the oviducts of *Dgcr8* ^md/md^ mice on Day 2. F, Representative images of 2‐cell embryos collected from the oviducts of *Dgcr8* ^f/f^ and *Dgcr8* cKO mice. Scale bar: 100 µm. G, Histological analyses of the ovaries of *Dgcr8* ^f/f^ and *Dgcr8* cKO mice collected on Day 2. * indicates corpus luteum, scale bar: 200 µm. Unpaired Student's *t* test, *P < 0.05, **P < 0.01

To explore the underlying causes of sub‐fertility in *Dgcr8* ^md/md^ mice, we examined in detail the reproductive phenotypes during early pregnancy. Since *Dgcr8* ^td/td^ mice are anovulatory due to pituitary defects, the numbers and fertilization rates of ovulated metaphase II (MII) oocytes were examined only in *Dgcr8* ^md/md^ and *Dgcr8* ^ed/ed^ mice. The number of ovulated MII oocytes was not statistically different between genotypes, although there was a moderate reduction in *Dgcr8* ^ed/ed^ and *Dgcr8* ^md/md^ mice (Figure 2A). However, the fertilization rate was significantly reduced in oocytes from *Dgcr8* ^md/md^ mice (Figure 2B). When 2‐cell embryos harvested from the *Dgcr8* ^md/md^ oviduct were cultured in vitro, they developed to the blastocyst stage similar to that of *Dgcr8* ^f/f^ and *Dgcr8* ^ed/ed^ mice (Figure 2C). We then investigated the quality and quantity of the oocyte right after ovulation at post‐hCG 16 hours. As shown in Figure 2D, the quantity and quality of oocytes were similar to those of *Dgcr8* ^f/f^ mice immediately after ovulation. This is consistent with the fact that zona pellucida remnants and degenerated oocytes were often observed from the oviducts of *Dgcr8* ^md/md^ mice on Day 2 (Figure 2E‐F). Furthermore, histological observation of the ovaries of *Dgcr8* ^md/md^ mice indicated that ovulation was not affected in these mice (Figure 2G).

### 3.4 Oviduct development was affected in *Dgcr8* ^md/md^ but not in *Dgcr8^td/td^* and *Dgcr8* ^ed/ed^ mice

**FIGURE 3:**
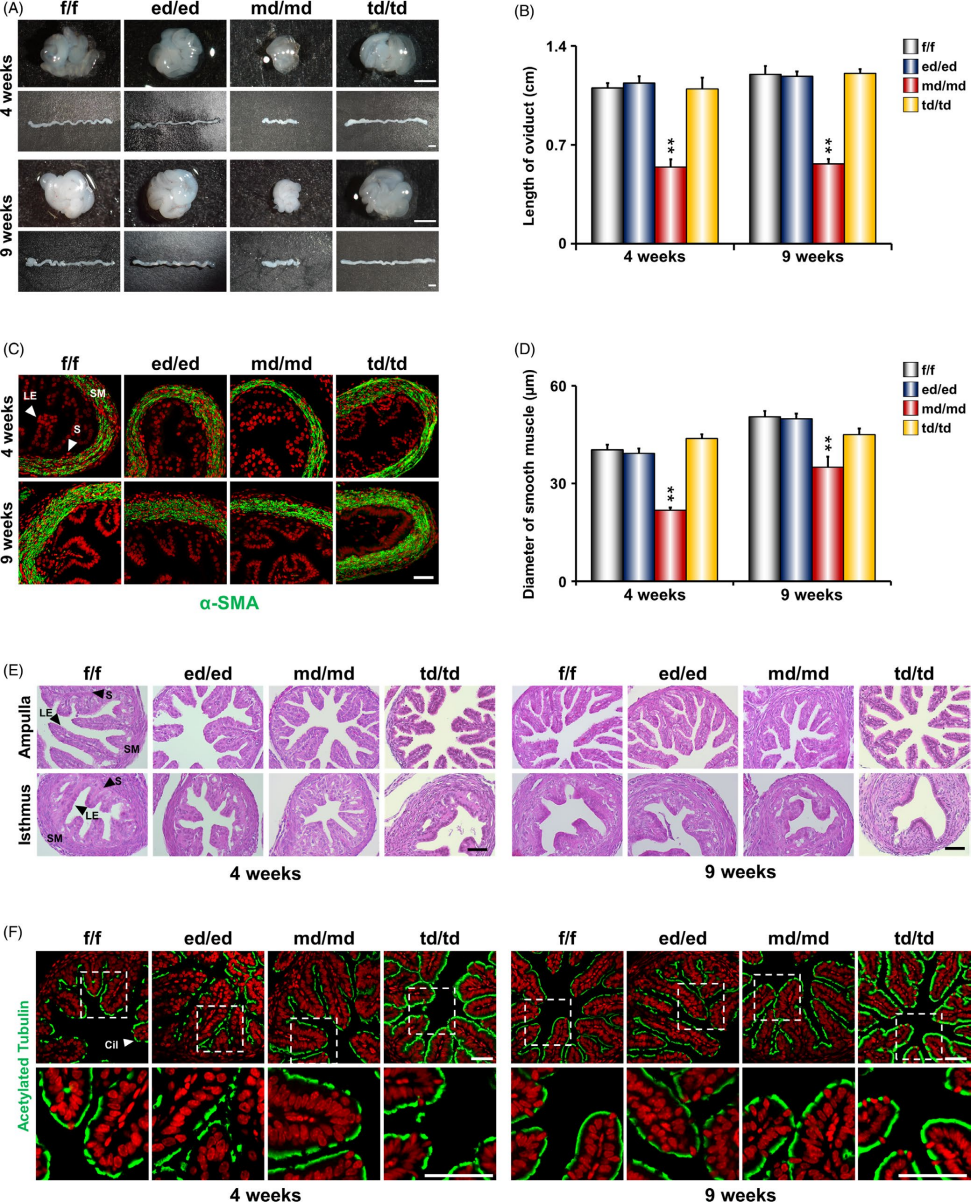
Gross and histological analyses in the oviducts of *Dgcr8* cKO mice at 4 and 9 weeks of ages. A, Gross morphology of the oviducts of *Dgcr8* ^f/f^ and *Dgcr8* cKO mice at 4 and 9 weeks of ages. Scale bar: 1 mm. B, Length of oviducts of *Dgcr8* ^f/f^ and *Dgcr8* cKO mice (n = 3‐7 per group). C, Immunofluorescence of α‐SMA in the isthmus sections from *Dgcr8* ^f/f^ and *Dgcr8* cKO mice. Scale bar: 50 µm. α‐SMA was visualized by green, and nuclei were stained with TO‐PRO‐3‐Iodide (red). D, Diameter of smooth muscle in the oviducts of *Dgcr8* ^f/f^ and *Dgcr8* cKO mice (n = 4‐6 per group). Smooth muscle thickness was determined by the length of the area of α‐SMA‐positive cell layers. E, Representative histological images of the oviducts of *Dgcr8* ^f/f^ and *Dgcr8* cKO mice at 4 and 9 weeks of ages. Scale bar: 50 µm. F, Immunofluorescence of acetylated Tubulin in the ampulla sections from *Dgcr8* ^f/f^ and *Dgcr8* cKO mice. Scale bar: 50 µm. Acetylated Tubulin was visualized by green, and nuclei were stained with TO‐PRO‐3‐Iodide (red). The bottom panels are high‐power images of the white window in the top panels. LE, Luminal epithelium; S, Stroma; SM, Smooth muscle; Cil, Cilia. Unpaired Student's *t* test, *P < 0.05, **P < 0.01

We next examined whether *Dgcr8* ^md/md^ mice showed any morphological abnormalities in the oviduct. The length of the oviduct in *Dgcr8* ^md/md^ mice was shorter than that in other *Dgcr8* cKO mice at 4 (pubertal) and 9 weeks (mature adult) of ages (Figure 3A‐B). Furthermore, the smooth muscle thickness of the isthmus in the oviduct was significantly reduced in both 4‐ and 9‐week‐old *Dgcr8* ^md/md^ mice, as observed using α‐SMA immunostaining (Figure 3C‐D). However, oviducts of all the genotypes*,* including *Dgcr8* ^md/md^ mice, were histologically indistinguishable from those of *Dgcr8* ^f/f^ mice and showed normal oviductal organization (Figure 3E). Furthermore, immunofluorescence staining for acetylated Tubulin was performed to examine whether the oviducts of *Dgcr8* cKO mice had cilia defects in the epithelial compartment (Figure 3F). The results were similar between oviducts of all the genotypes of *Dgcr8* cKO mice, suggesting that the distribution and function of oviductal cilia of *Dgcr8* cKO mice were not different from those of *Dgcr8* ^f/f^ mice.

### 3.5 Deletion of *Dgcr8* affects the uterine architecture with Cre‐specific distinct spectrum

**FIGURE 4:**
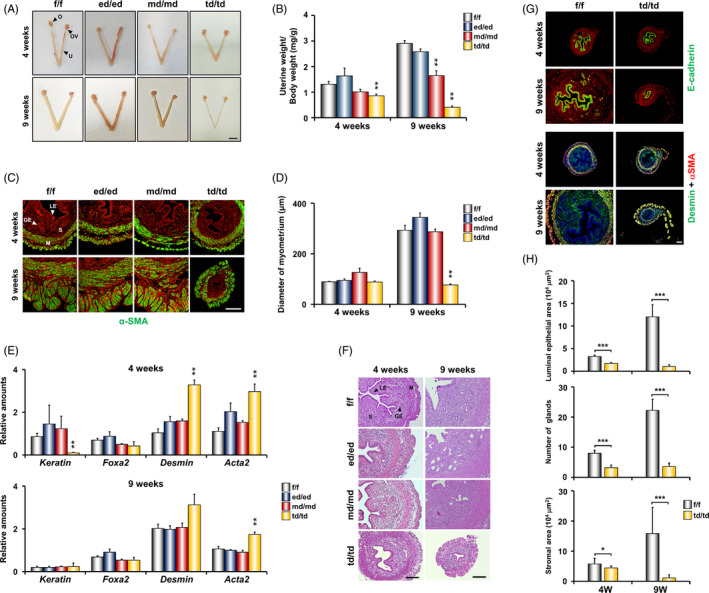
Gross and histological analyses of uterus in *Dgcr8* cKO mice. A, Gross morphology of female reproductive tracts in *Dgcr8* ^f/f^ and *Dgcr8* cKO mice at 4 and 9 weeks of ages. Scale bar: 5 mm. B, Changes in uterine weight/total body weight of *Dgcr8* ^f/f^ and *Dgcr8* cKO mice (n = 4‐11 per group). C, Immunofluorescence of α‐SMA in the uterine sections from *Dgcr8* ^f/f^ and *Dgcr8* cKO mice. α‐SMA was visualized by green, and nuclei were stained with TO‐PRO‐3‐Iodide (red). Scale bar: 50 µm. D, Myometrial thickness in the uteri of *Dgcr8* ^f/f^ and *Dgcr8* cKO mice (n = 4‐6 in each group). Myometrial thickness was determined by the length of the area of α‐SMA‐positive cell layers. E, Real‐time RT‐PCR analyses of relative mRNA levels of the cell type marker genes (*Keratine*; Epithelial cell, *Foxa2;* Glandular epithelial cell, *Desmin;* Stromal cell, and *Acta2;* Smooth muscle cell) in the uterus of *Dgcr8* ^f/f^ and *Dgcr8* cKO mice (n = 4 per group). F, Histological analyses of the uterine sections in *Dgcr8* ^f/f^ and *Dgcr8* cKO mice. Scale bar: 100 µm. G, Immunofluorescence of E‐cadherin (Epithelial cell; green), Desmin (Stromal cell and myometrium; green) and α‐SMA (Smooth muscle cell; red) in the uterine sections from *Dgcr8* ^f/f^ and *Dgcr8* ^td/td^ mice. Nuclei were stained with DAPI (blue) and TO‐PRO‐3 (red). Scale bar: 50 µm. H, Luminal epithelial area (Top panel), number of glands (Middle panel), and Stromal area (Bottom panel) in the uteri of *Dgcr8* ^f/f^ *and Dgcr8* ^td/td^ mice at 4 and 9 weeks of age (n = 3‐7 in each group). O, Ovary; OV, Oviduct; U, Uterus; GE, Glandular epithelium; LE, Luminal epithelium; S, Stroma; M, Myometrium. Unpaired Student's *t* test, *P < 0.05, **P < 0.01

To examine whether *Dgcr8* deficiency affects uterine development, we investigated the uteri of all the *Dgcr8* cKO mice at 4 and 9 weeks of ages. The gross morphology and uterine weight to body weight ratio of 4‐week‐old *Dgcr8* ^td/td^ mice were different from all the other genotypes. However, at 9 weeks of age, these defects were observed not only in *Dgcr8* ^td/td^ mice, but also in *Dgcr8* ^md/md^ mice (Figure 4A‐B). Interestingly, a severely atrophic myometrium was observed only in adult *Dgcr8* ^td/td^ mice (Figure 4C‐D). In general, gross histology and real‐time RT‐PCR analysis and/or immunostaining of cell type‐specific markers showed that *Dgcr8* ^ed/ed^ and *Dgcr8* ^md/md^ uteri were similar to those of *Dgcr8* ^f/f^ mice (Figure 4C‐F), whereas *Dgcr8* ^td/td^ uteri had severe abnormalities, such as reduced myometrial thickness, and reduced stromal and epithelial area (Figure 4G‐H).

### 3.6 mRNAs that control immune responses and negatively regulate smooth muscle cell proliferation were systemically upregulated in the uteri of *Dgcr8* cKO mice

**FIGURE 5:**
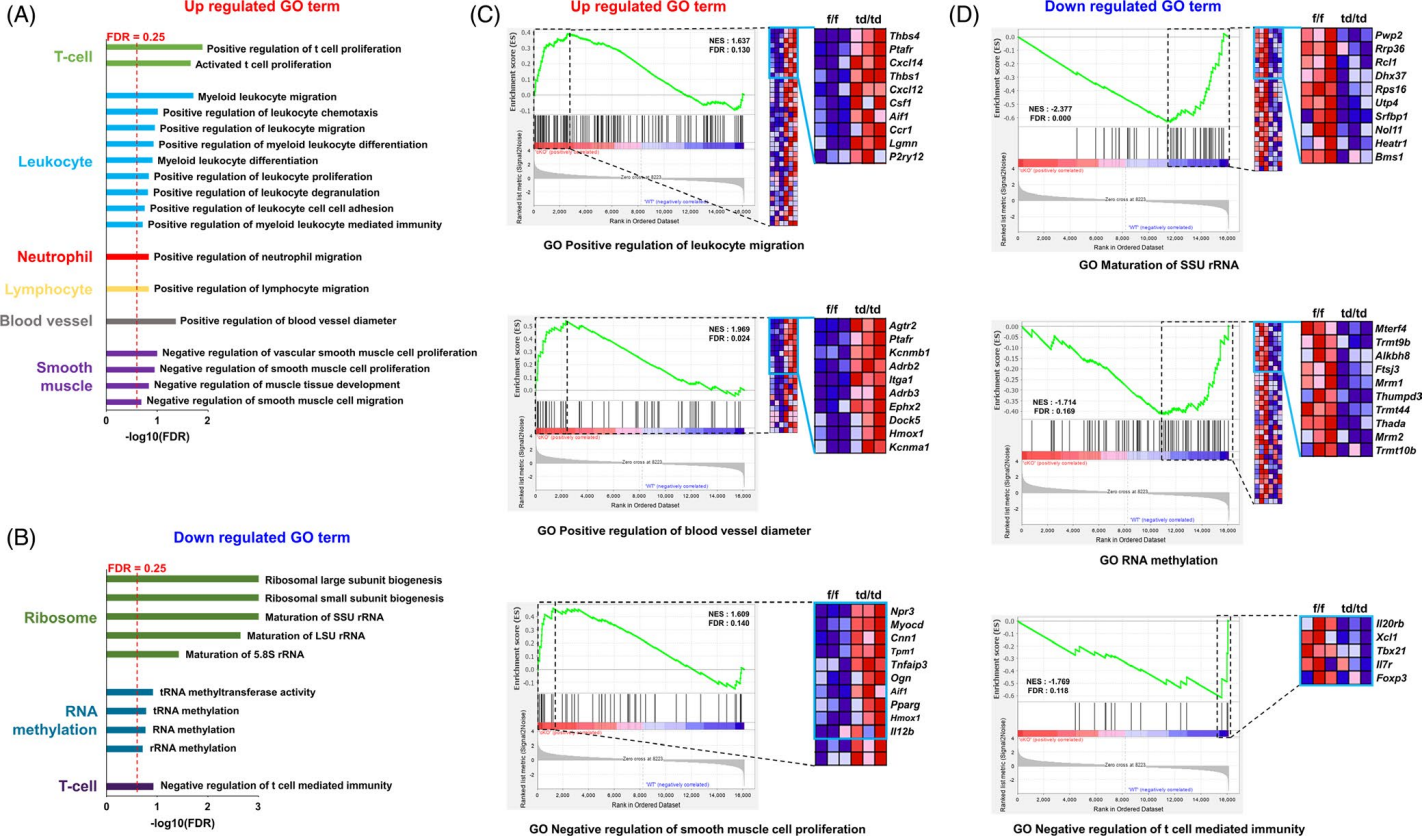
GSEA analysis revealed upregulated and downregulated gene sets via mRNA seq in the uterus of *Dgcr8* ^td/td^ at 4 weeks of age. (A, B) GSEA was performed to identify upregulated (A) and downregulated (B) gene ontology (GO) term in *Dgcr8* ^td/td^ mice at 4 weeks of age. Gene sets with an FDR q‐value < 0.25 (red dotted line) were considered significant. (C, D) GSEA enrichment plots and heatmaps of upregulated (C) and downregulated (D) GO gene sets from RNA‐seq data. The normalized enrichment score (NES) and the corresponding FDR q‐value are reported in each graph

**FIGURE 6:**
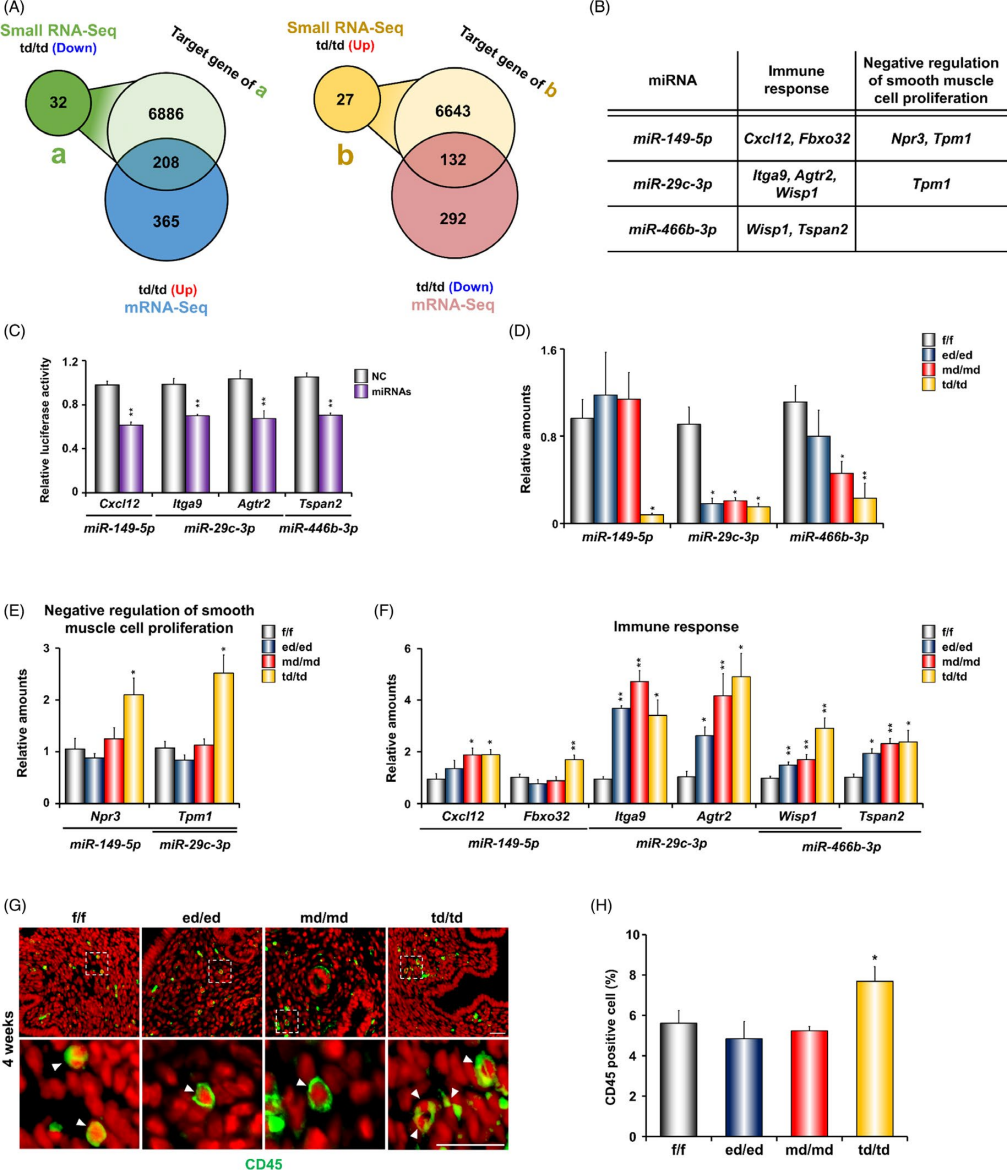
Expression of potential target genes related to immune response and negative regulation of smooth muscle proliferation in the uteri of *Dgcr8* cKO mice at 4 weeks of age. A, Venn diagram indicates overlapping between downregulated miRNA (a; 32, *P* < 0.05, 1.5‐fold change) target genes (n = 7094) and upregulated genes (n = 573), and upregulated miRNA (b; 27, *P* < 0.05, 1.5‐fold change) target genes (n = 6775) and downregulated genes (n = 424) in *Dgcr8* ^td/td^ mice at 4 weeks of age. The target genes of miRNA were searched using miRmap. B, Potential target genes for miRNAs related to immune response and negative regulation of smooth muscle proliferation in the uterus of *Dgcr8* ^td/td^ mice at 4 weeks of age. C, Luciferase reporter assay of *Dgcr8* potential target miRNAs binding to 3’ UTR of putative target genes. (D‐F) Real‐time RT‐PCR analyses of relative miRNAs and their targeted mRNA levels in the uterus of *Dgcr8* ^f/f^ and *Dgcr8* cKO mice. (n = 4‐8 per group). G, Immunofluorescence of CD45 in the uterine sections from *Dgcr8* ^f/f^ and *Dgcr8* cKO mice. CD45 (immune cell marker) was visualized by green, and nuclei were stained with TO‐PRO‐3 (red). Scale bar: 20 µm. H, Percentage of CD45‐positive cell /total number of cells counted in the uteri of *Dgcr8* ^f/f^ and *Dgcr8* cKO mice at 4 weeks of age (n = 10‐11 in each group). Unpaired Student's *t* test, *P < 0.05, **P < 0.01

We then performed small RNA‐seq and mRNA‐seq for uteri of 4‐week‐old *Dgcr8^td/td^* mice to elucidate the molecular mechanisms underlying the severe uterine phenotypes (Figure 5). Uteri from 4‐week‐old *Dgcr8* ^td/td^ mice were chosen because they showed the onset of multiple uterine defects*,* and *PR* was also expressed in all the major uterine cell types at this stage.^4^ In mRNA‐seq data, 573 and 424 genes with 1.5‐fold cut‐off values were upregulated and downregulated in *Dgcr8* ^td/td^ mice, respectively (Figure S2 and Table S2). GSEA analyses showed the systemic upregulation of the gene sets associated with ‘immune response’, including leucocyte proliferation, migration, chemotaxis and blood vessel dilation (Figure 5A, C). In addition, gene sets associated with ‘negative regulation of smooth muscle cell proliferation and development’ were upregulated (Figure 5A, C). These results are consistent with the phenotypes in *Dgcr8* ^td/td^ mice, such as acute inflammatory infiltration of immune cells in the female reproductive tract and severe atrophy in uterine smooth muscle (Figures 4 and 6). In contrast, gene sets involved in ribosome biogenesis, RNA methylation (RNA stability)^17^ and negative regulation of T cell‐mediated immunity were mainly downregulated in *Dgcr8* ^td/td^ mice (Figure 5B, D). Small RNA‐seq data showed that 1035 out of the 1976 mouse miRNAs were detected in the uterus. However, only 32 and 27 miRNAs were down‐ and upregulated, respectively, with a 1.5‐fold cut‐off value in *Dgcr8* ^td/td^ mice (Figure S3 and Table [Link], [Link]).

### 3.7 *Dgcr8* cKO mice provide distinct and overlapped target profiles in a Cre‐dependent manner

To identify the direct target mRNAs of differentially expressed miRNAs (DEMs) in the uteri of *Dgcr8* ^td/td^ mice, we obtained a dataset of potential target genes of DEMs in *Dgcr8* ^td/td^ mice using miRmap and compared this dataset with mRNA‐seq data. We found that 208 upregulated and 132 downregulated genes were overlapped between both datasets (Figure 6A). Since *miR‐149‐5p, miR‐29c‐3p* and *miR‐446b‐3p* miRNAs are representative of miRNAs with a Cre‐specific unique expression in the uterus, these miRNAs and their target mRNAs associated with ‘immune response’ and/or ‘negative regulation of smooth muscle cell proliferation and development’ were further evaluated (Figure 6B). When luciferase constructs that included the 3’ UTR of *Cxcl12*, *Agtr2*, *Itga9* or *Tspan2* mRNAs were co‐transfected with miRNA mimics, the luciferase activity was significantly reduced (Figure 6C). This suggests that the target mRNAs that control the immune response are directly regulated by the miRNAs.

We further examined and compared the expression profiles of these miRNAs and their target genes between *Dgcr8* cKO mice with different Cre systems. *miR‐29c‐3p* was reduced in all the cKO genotypes and *miR‐466b‐3p* was differentially regulated in a Cre‐specific manner, whereas *miR‐149‐5p* was significantly reduced only in *Dgcr8* ^td/td^ mice (Figure 6D). In general, the unique expression profiles of these miRNAs were inversely correlated with their target mRNAs in the uterus (Figure 6E,F). For example, among the *miR‐149‐5p* targets, *Fbxo32*, *Npr3* and *Tpm1* were specifically upregulated only in the *Dgcr8* ^td/td^ uterus, where *miR‐149‐5p* was significantly reduced. *Cxcl12* was upregulated not only *in Dgcr8* ^td/td^ but also in the *Dgcr8* ^md/md^ uteri. Furthermore, the expression patterns of *Itga9* and *Agtr2*, targets of *miR‐29c‐3p*, were inversely upregulated in all the three *Dgcr8* cKO mice. *Wisp1*, a target of *miR‐29c‐3p* and *miR‐466b‐3p*, were inversely correlated with their regulator miRNAs. The upregulation of *Npr3* and *Tpm1* as well as downregulation of their regulator *miR‐149‐5p* only in *Dgcr8* ^td/td^ uterus (Figure 6E) were consistent with the fact that smooth muscle defects were observed only in the *Dgcr8* ^td/td^ uterus (Figure 4D). Interestingly, whereas miRNAs and their target mRNAs associated with immune response were differentially dysregulated in *Dgcr8* cKO mice in a Cre‐dependent manner (Figure 6F), the percentage of CD45‐positive immune cells were significantly increased only in *Dgcr8* ^td/td^ mice at 4 weeks of ages (Figure 6G,H). Interestingly, the acute immune infiltration into the organs in the female reproductive tract was persistently observed in the *Dgcr8* ^td/td^ uterus, but not in other genotypes (Figure S4).

## 4 DISCUSSION

The uterus is a complex organ that consists of three major tissue compartments: myometrium, stroma and epithelium, with dynamic changes in various immune cells during the reproductive cycle. In this aspect, *PR* is a good Cre driver given its expression in all the major uterine cell types, and thus, *PR‐*Cre mice have been exploited for gene deletion studies in all the major uterine compartments.^5^, ^18^ Recently, we also demonstrated that canonical miRNAs are essential for uterine physiology and fertility using *Dgcr8* ^td/td^ mice.^4^ However, *PR‐*Cre mice may display compound phenotypes in multiple cell types. Thus, comparative analyses of all the three *Dgcr8* cKO mice in this study will improve the understanding of the spatiotemporal action of each Cre system in the female reproductive tract. When *Dgcr8* is deleted by *Amhr2*‐Cre, defects in the oviduct and uterus were observed before and after puberty, respectively (Figures 3 and 4). These results suggest that phenotypes are exposed much later than the onset of Cre expression and/or follow a tissue‐specific expression in *Dgcr8* ^md/md^ mice. Unlike *Dgcr8* ^td/td^ mice that suffer from infertility and uterine deformities, *Dgcr8* ^ed/ed^ and *Dgcr8* ^md/md^ mice showed regular oestrous cycles, showed normal architecture, and produced pups, suggesting that the deletion of *Dgcr8* in epithelial and mesenchymal cells does not significantly deteriorate fertility (Figure 1). However, considering that both *PR* and *Amhr2* are expressed in the stroma and myometrium in the female reproductive tract,^19^ it is noteworthy that *Dgcr8* ^td/td^, but not *Dgcr8* ^md/md^ mice exhibited acute inflammation in all the tissues of the female reproductive tract (Figure 6 and Figure S4). These results suggest that immune cells deficient in canonical miRNAs may affect uterine development and physiology. In fact, *PR* is present in a variety of cell types, including immune cells, such as NK cells,^6^ macrophages,^7^ dendritic cells^8^ and T cells,^9^ and non‐immune cells, such as neuronal cells. However, it was also reported that *Ltf* is expressed in some immune cells, especially neutrophils and macrophages.^12^, ^16^ Thus, deletion of *Dgcr8* using immune‐cell‐specific Cre, such as macrophage‐specific *LyzM*‐Cre,^20^ may provide clues to decipher these puzzled phenotypes that are observed in *Dgcr8* ^td/td^ mice but not in others.

**FIGURE 7:**
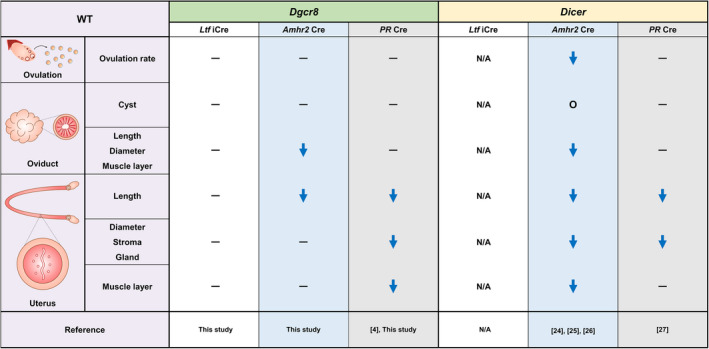
Phenotypic comparison of *Dgcr8* cKO and *Dicer* cKO mice with three Cre systems. ━, Normal; ↓, Decrease; N/A, Not available

DGCR8 is required for the biogenesis of canonical miRNAs, whereas DICER is indispensable in the production of both canonical and non‐canonical miRNAs. Thus, it is expected that *Dicer* and *Dgcr8* cKO mice would show overlapping phenotypes in the female reproductive tract. In fact, *Dgcr8* ^md/md^ mice showed similar but less severe abnormalities compared to *Dicer* ^md/md^ mice. For example, *Dgcr8* ^md/md^ and *Dicer* ^md/md^ mice showed abnormally short oviducts, whereas *Dgcr8* ^td/td^ and *Dicer* ^td/td^ mice did not display morphologic deformities in the oviduct (Figure 7). While embryos were trapped in the oviduct that often harboured cysts during early pregnancy in *Dicer* ^md/md^ mice,^21^, ^22^, ^23^ but not in *Dgcr8* ^md/md^ mice (Figure 2), the increased number of degenerated oocytes in the oviduct of *Dgcr8* ^md/md^ mice could be associated with this phenotype. The uterus of *Dicer* ^md/md^ mice showed morphological and histological defects^21^, ^22^, ^23^ whereas *Dgcr8* ^md/md^ mice had a normal uterine structure (Figure 4). In contrast, when *PR‐*Cre was used, *Dgcr8* ^td/td^ and *Dicer* ^td/td^ mice showed similar uterine phenotypes, such as decreased number of glands and atrophic stroma.^24^ In addition, considering that *Dgcr8* is an essential factor for the biogenesis of canonical miRNAs, it was quite surprising that only 32 miRNAs with a 1.5‐fold change were downregulated in the uteri of *Dgcr8* ^td/td^ mice (Figure 6). Intriguingly, only a handful of miRNAs were downregulated in the uteri of *Dicer* ^td/td^ mice as well.^24^

*PR*‐Cre, *Amhr2*‐Cre and *Ltf*‐iCre mice have been employed to understand the functions of other genes important for uterine biology. For example, each Cre mouse was individually used to examine the function of *Pten*, a well‐known tumour suppressor gene, in tumorigenesis of endometrial cancer.^25^, ^26^, ^27^, ^28^ *Pten* ^td/td^ mice showed rapid development of endometrial cancer with full penetration, whereas *Pten* ^md/md^ mice failed to initiate tumorigenesis. As *Amhr2* is not expressed in the epithelial compartment of the uterus, it is reasonable that *Pten* deletion in the stroma and myometrium could not provoke endometrial cancer.^25^, ^26^ However, *Pten* ^ed/ed^ mice developed atypical epithelial hyperplasia but did not develop endometrial cancer,^27^ suggesting that *Pten* signalling in the stroma restrains epithelial cell transformation from hyperplasia to carcinoma. Deletion of *Tsc1*, a direct inhibitor of mTORC1*,* in the female reproductive tract sterilized both *Tsc1* ^td/td^ and *Tsc1* ^md/md^ female mice, resulting from oviductal hyperplasia, retention of embryos in the oviduct, and implantation failure.^19^ However, embryo development was disrupted in *Tsc1* ^td/td^, but not in *Tsc1* ^md/md^ mice.^19^ Collectively, these reports, as well as this study, suggest that selection of the Cre system leads to a differential spectrum of phenotypes in tissues with multiple cell types in the female reproductive tract.

Previously, we demonstrated that DGCR8‐dependent canonical microRNAs are essential for uterine development and physiological processes such as proper immune modulation, reproductive cycle and steroid hormone responsiveness in mice.^4^ Especially, we observed that an excessive influx of immune cells occurs in the ovary, oviduct and uterus of *Dgcr8* ^td/td^ mice on Day 2.^4^ In addition, percentage of CD45‐positive cells were increased in the uterus of 4‐week‐old *Dgcr8* ^td/td^ mice (Figure 6G, H). These are consistent with the results of small RNA‐ and mRNA‐seq, which revealed that miRNAs and their potential target mRNAs involved in immune responses were dysregulated in the uteri of *Dgcr8* ^td/td^ mice. However, acute inflammation in *Dgcr8* ^td/td^ mice was not observed in *Dgcr8* ^md/md^ and *Dgcr8* ^ed/ed^ mice (Figure 6 and Figure S4), suggesting that the profiles of dysregulated miRNAs could depend on the Cre system. Although many miRNAs are known to regulate immune responses, such as Toll‐like receptor (TLR) signalling,^29^ the functions of these miRNAs in the uterus are poorly understood. Luciferase assays validated that miRNAs directly inhibit *Cxcl12*, *Itga9, Agtr2* and *Tspan2* (Figure 6C). ITGA9 plays a very important role in neutrophil migration,^30^ and CXCL12 induces cell migration via the CXCR4/CXCR7 complex.^31^, ^32^, ^33^ TSPAN2 induces M2 polarization in microglia, and AGTR2 has vasodilating and blood pressure‐reducing effects.^34^, ^35^ Another unique phenotype observed in *Dgcr8* ^td/td^ uteri was that the inner circular smooth muscle became atrophic at the adult stage (Figure 4). The role of miRNAs in the proliferation of uterine smooth muscle cells is unknown, but *Npr3* and *Tpm1*, potential target genes of *miR‐149‐5p* and *miR‐29c‐3p*, are known to negatively regulate smooth muscle cell proliferation. NPR3 increases endothelial cell proliferation and inhibits vascular smooth muscle growth via ERK 1/2 phosphorylation.^36^ In addition, TPM1 inhibits vascular smooth muscle proliferation and migration progression via the HIF‐1α/ *miR‐21* / TPM1 pathway.^37^ Although the expression of *miR‐21* was not reduced in *Dgcr8* ^td/td^ mice, it is thought that the expression could be sufficiently regulated by other miRNAs. Thus, it is assumed that the downregulation of miRNAs maintains the increased levels of their target mRNAs, subsequently inducing leucocyte migration and differentiation, blood vessel dilation, and/or suppression of smooth muscle proliferation. Collectively, differential phenotypes and landscapes of miRNAs and mRNAs in *Dgcr8* cKO mice with different Cre systems suggest that selection of Cre is critical to understanding the function of the gene of interest in the female reproductive tract in a spatiotemporal manner.

## CONFLICT OF INTEREST

The authors declare no competing financial interests.

## AUTHOR CONTRIBUTIONS

YS Kim, SC Yang, HJ Lim and H Song conceived and designed the experiments. YS Kim, SC Yang, M Park and HR Kim carried out the experiments. YS Kim, SC Yang, M Park, Y Choi, FJ DeMayo, JP Lydon, HJ Lim and H Song analysed the data. YS Kim, SC Yang, HJ Lim and H Song wrote the manuscript. All authors agreed to be responsible for the content of the work.

## Supporting information

Figure S1

Figure S2

Figure S3

Figure S4

Table S1

Table S2

Table S3

Table S4

## Data Availability

The data that support the findings of this study are available from the corresponding author upon reasonable request.
