# Supplementary figures and images for: Different Cre systems induce differential microRNA landscapes and abnormalities in the female reproductive tracts of Dgcr8 conditional knockout mice
Source: Cell Prolif. 2021 Jan 26;54(3):e12996. doi: 10.1111/cpr.12996 (PMC7941225; doi:10.1111/cpr.12996)

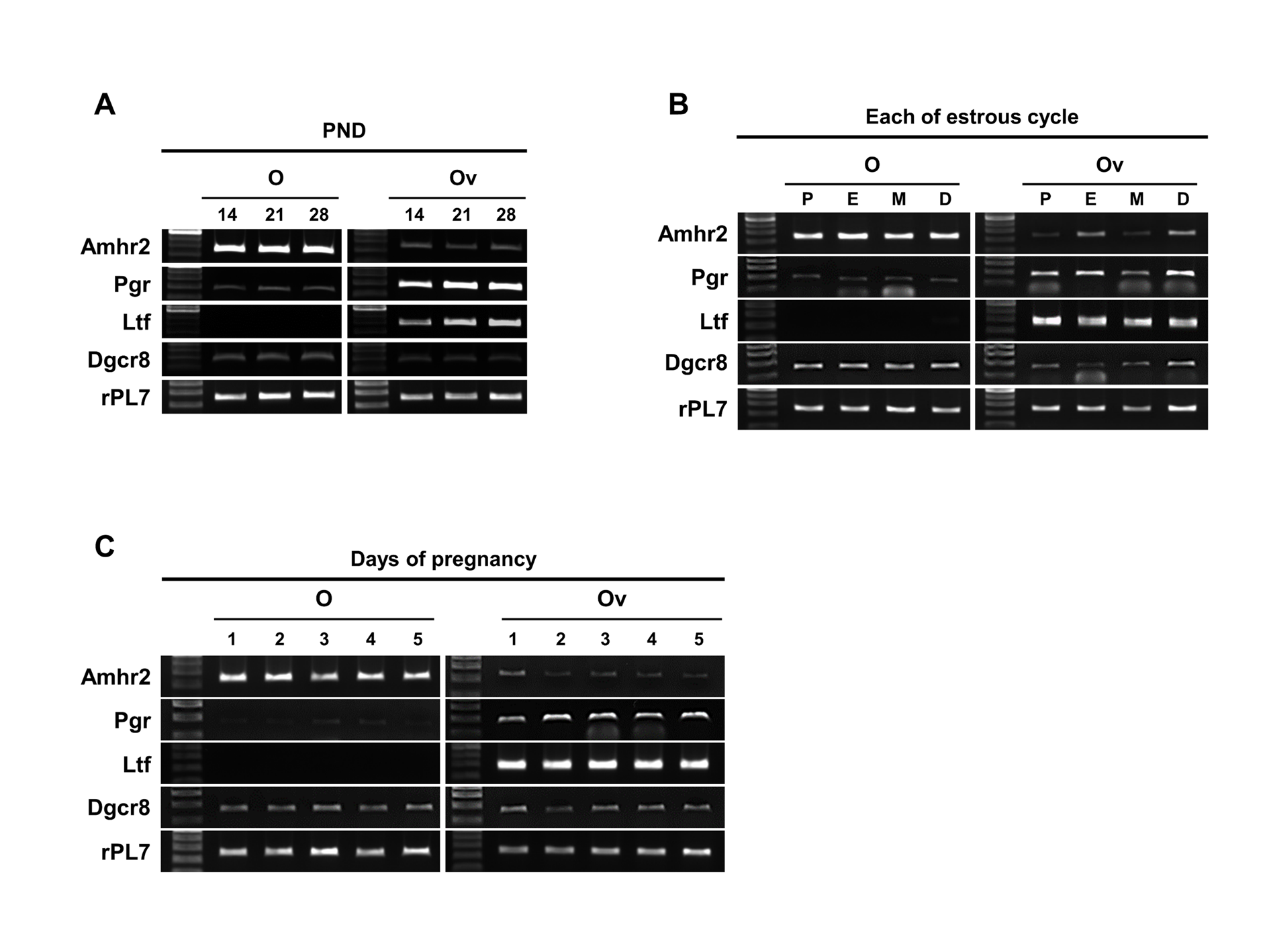

Supplement: Supplementary file 1 — Figure S1 [file CPR-54-e12996-s003.tif]

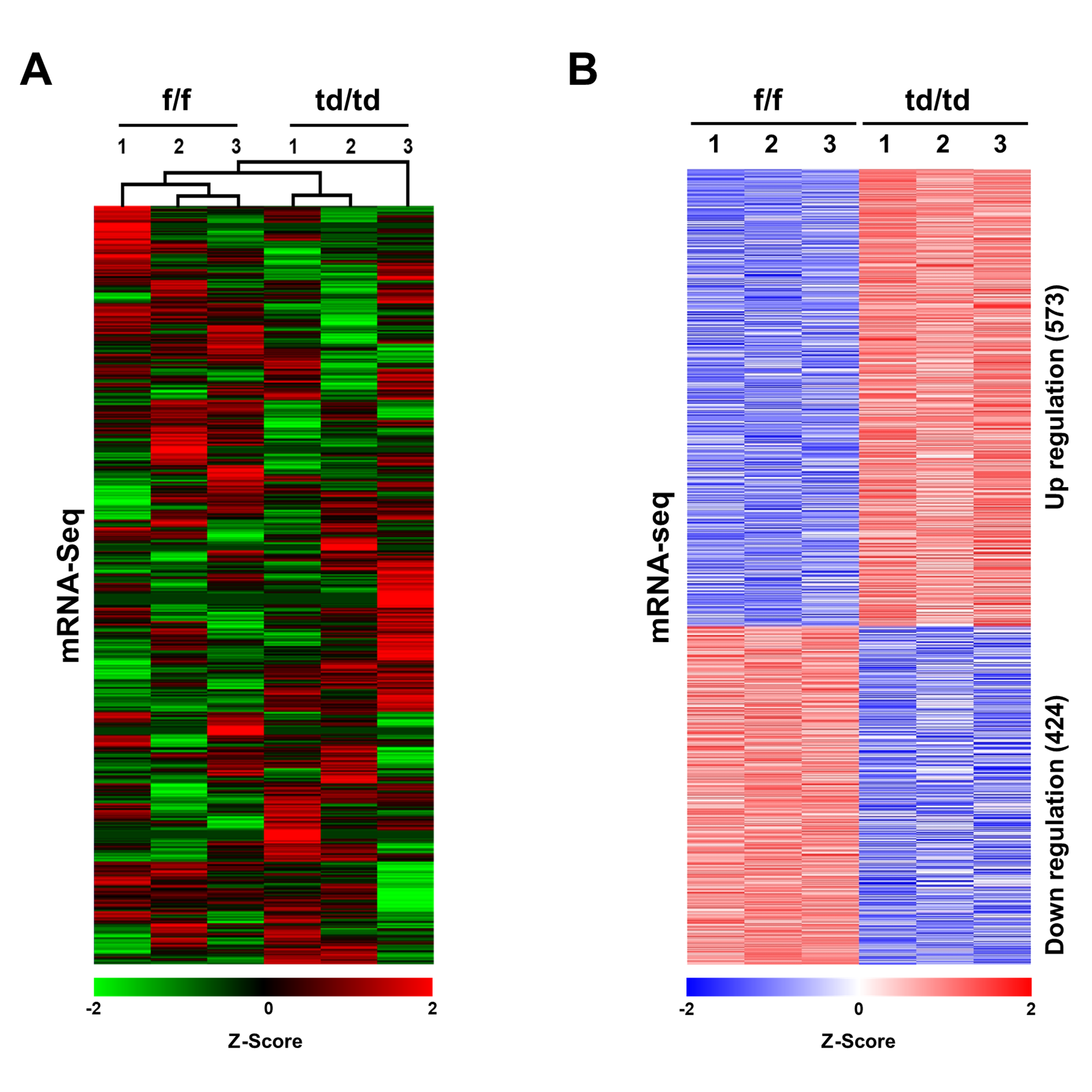

Supplement: Supplementary file 2 — Figure S2 [file CPR-54-e12996-s008.tif]

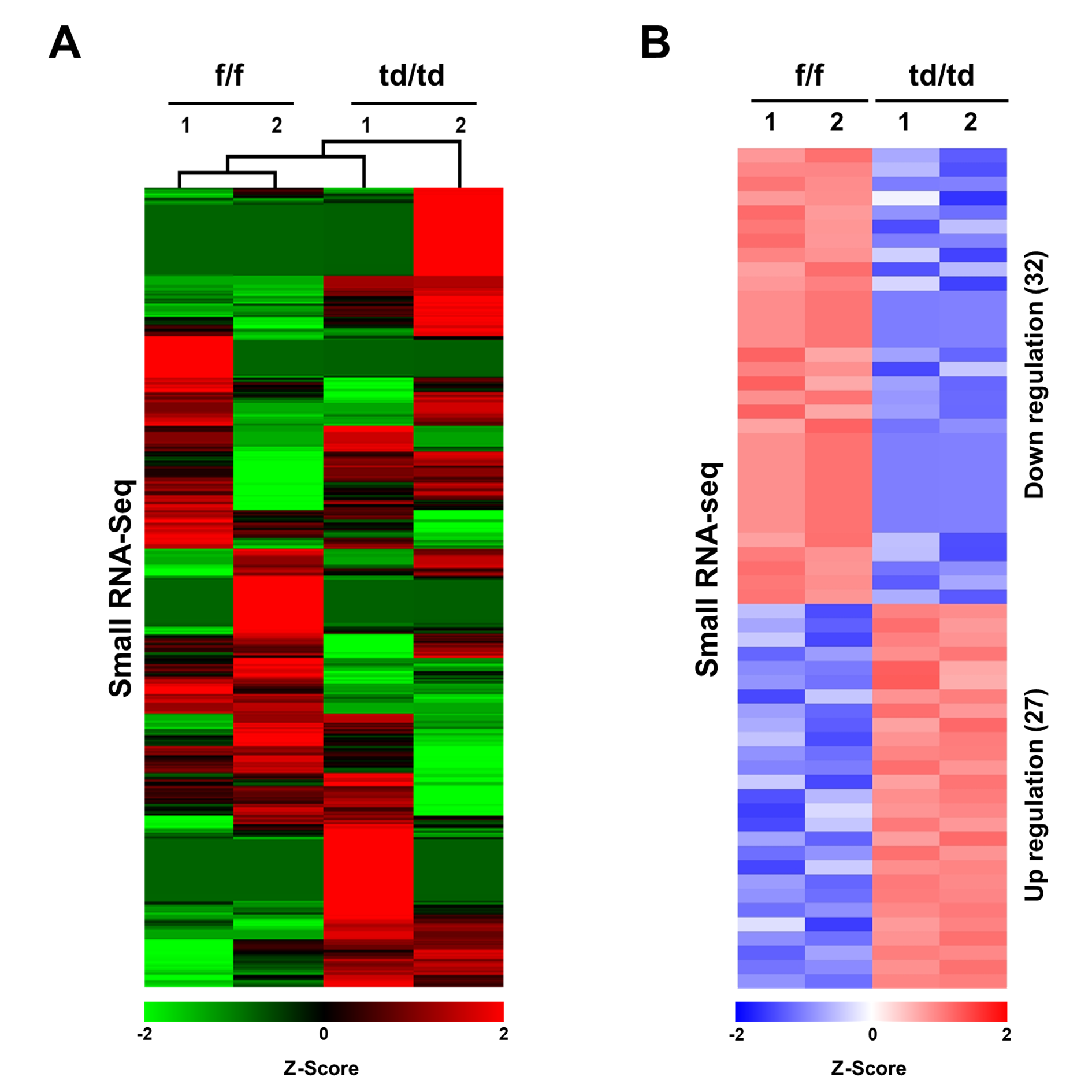

Supplement: Supplementary file 3 — Figure S3 [file CPR-54-e12996-s001.tif]

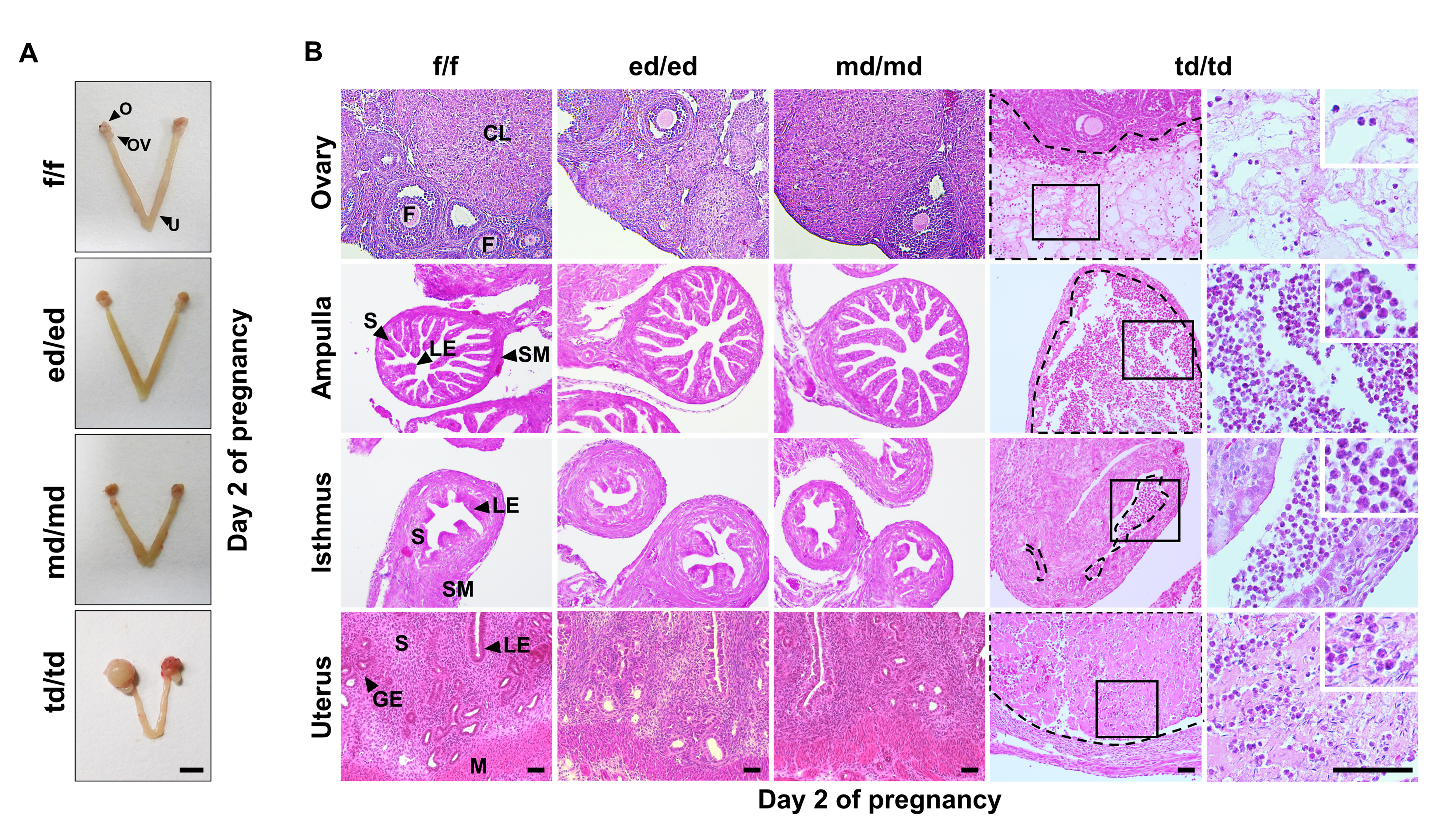

Supplement: Supplementary file 4 — Figure S4 [file CPR-54-e12996-s006.tif]
